# Preoperative photocoagulation reduces corneal endothelial cell damage after vitrectomy in patients with proliferative diabetic retinopathy

**DOI:** 10.1097/MD.0000000000007971

**Published:** 2017-10-27

**Authors:** Jie Zhong, Jun Jia, Jiguo Yu, Liping Zhang, Yi Xiang

**Affiliations:** Department of Ophthalmology, Key Laboratory for Molecular Diagnosis of Hubei Province, The Central Hospital of Wuhan, Tongji Medical College of Huazhong University of Science and Technology, Wuhan, Hubei Province, China.

**Keywords:** corneal endothelial cells, preoperative photocoagulation, proliferative diabetic retinopathy, vitrectomy

## Abstract

Proliferative diabetic retinopathy (PDR) is a severe complication of diabetes and is a leading cause of visual decline and irreversible blindness. So we designed this study to investigate retrospectively the effect of preoperative photocoagulation on corneal endothelial cells after vitrectomy in patients with PDR.

The study included 52 eyes of 46 patients with PDR complicated with vitreous hemorrhage, who underwent vitrectomy. Patients were apportioned to a photocoagulation group (26 eyes/23 patients) or nonphotocoagulation group (26/23 patients), according to their history of preoperative photocoagulation. A specular microscope was used to assess the corneal endothelial cell density and percentage of hexagonal cells (PHC) before surgery, and at 1 week, 1 month, and 3 months after surgery.

The cell density was lower 3 months after surgery in the photocoagulation group, but at 1 month in the nonphotocoagulation group, all cases were significantly different from the preoperative value (*P* < .05 or *P* < .01). One week after surgery, the mean cell densities between the photocoagulation and nonphotocoagulation groups were not statistically different (*P* > .05). However, the mean cell densities at 1 and 3 months after surgery in the photocoagulation group were significantly higher than those in the nonphotocoagulation group (*P* < .05). The PHC values in the photocoagulation group at 1 week and in the nonphotocoagulation group at 1 week, 1 month, and 3 months were much lower than their respective preoperative values (*P* < .05 or *P* < .01). More importantly, at 1 and 3 months, the PHC had recovered to preoperative values in the photocoagulation group, but not in the nonphotocoagulation group. As for cell density and PHC, they were both significantly higher 1 and 3 months after surgery in the photocoagulation group than in the nonphotocoagulation group (*P* < .05).

Photocoagulation before vitrectomy reduces subsequent corneal endothelial cell damage in PDR patients.

## 1 Introduction

Proliferative diabetic retinopathy (PDR) is a severe complication of diabetes, characterized by the growth of new blood vessels.^[1]^ PDR can result in vitreous hemorrhage, neovascular membranes, and tractional retinal detachment, which is a leading cause of visual decline and irreversible blindness.^[2]^ Laser photocoagulation is an effective method to prevent the progression of PDR and reduce PDR-induced blindness.^[3]^ However, retinopathy can progress despite adequate photocoagulation and systemic control of blood glucose, lipid, and cholesterol levels. To restore vision, vitrectomy is often required for PDR patients, especially for those with vitreous hemorrhage and retinal detachment.^[4]^ Although the visual outcome after vitrectomy varies greatly, most patients benefit.^[5,6]^

Corneal endothelial cells are nonregenerating hexagonal cells that have an important role in maintaining corneal transparency,^[7]^ but are subject to damage by surgical trauma such as in cataract surgery^[8,9]^ and excimer laser keratorefractive surgery.^[10]^ Diabetes is putatively associated with structural and functional changes in the corneal endothelial cell.^[11–14]^ Several studies have reported that in diabetic patients, the morphology of corneal endothelial cells is susceptible to change during cataract surgery, and patients are at a high risk of corneal endothelial cell loss afterward.^[15,16]^ Furthermore, patients with diabetic retinopathy are at increased risk of corneal decompensation after cataract surgery.^[17]^ Therefore, diabetes and surgical trauma are risk factors for corneal endothelial cell loss.

It has been reported that vitrectomy also can result in corneal endothelial cell loss.^[18,19]^ Goezinne et al^[20]^ reported that corneal endothelial cell loss was found after vitrectomy with silicone oil in patients with complex retinal detachment. However, the effect of vitrectomy on corneal endothelial cells in PDR patients has not been investigated.

In this study, we retrospectively analyzed 52 eyes of 47 PDR patients complicated with vitreous hemorrhage, who underwent vitrectomy. The purpose of this study was to investigate the effect of photocoagulation on corneal endothelial cells in PDR patients, relative to no photocoagulation treatment before vitrectomy.

## 2 Materials and methods

### 2.1 Patients

The Medical Ethics Committee of the Central Hospital of Wuhan approved this retrospective study, and all patients gave their written informed consent. The study included 52 eyes of 46 patients with PDR (stage IV–V) complicated with vitreous hemorrhage, who underwent vitrectomy at our hospital between March 2011 and June 2015. Twenty-four patients (28 eyes) were men and 22 patients (24 eyes) were women. The mean age was 60.6 years (range, 24–75 years).

The inclusion criteria were: patients with Type I or Type II diabetes; reduced vision with visual acuity ranging between hand motion and 0.1; PDR diagnosed by ophthalmologic examination, B-ultrasonography, optical coherence tomography, and fundus fluorescein angiography; one or both eyes treated with vitrectomy; normal preoperative intraocular pressure (IOP); and lack of severe postoperative complications.

Patients with the following were excluded from this study: ocular diseases such as corneal lesion, ocular trauma, glaucoma, or retinal detachment; history of vitrectomy; and eye-related systemic diseases such as thyroid-associated ophthalmopathy.

Patients were apportioned to 2 groups according to the history of preoperative photocoagulation as follows: photocoagulation group (26 eyes of 23 patients) and nonphotocoagulation group (26 eyes of 23 patients). For patients in the photocoagulation group, the mean disease duration of diabetes was 13.3 years (range, 4–30 years). Two eyes had a disease duration ≤5 years, 8 eyes had a disease duration between 6 and 10 years, and 16 eyes had a disease duration >10 years. For patients in the nonphotocoagulation group, the mean disease duration of diabetes was 14.5 years (range, 4–25 years). Three eyes had a disease duration ≤5 years, 6 eyes had a disease duration between 6 and 10 years, and 17 eyes had a disease duration >10 years.

For patients in the photocoagulation group, the time between the first photocoagulation and vitrectomy ranged from 1 month to 2 years, including ≤3 months in 5 eyes, between 4 and 12 years in 13 eyes, and >1 year in 8 eyes. At the time of the first photocoagulation, 7 eyes had severe non-PDR, and 19 eyes had PDR. Pan-retinal photocoagulation was performed with laser spots located >1 pupillary distance (PD) nasal to the optic disc, >1 PD superior and inferior to the optic disc, and >2 PD temporal to the macula extending to the equator. Photocoagulation was performed in 4 sessions with each quadrant per session. The between-session interval was 1 week. Each treatment consisted of 300 to 500 burns. All the treatments were performed by the same ophthalmologist.

### 2.2 Vitrectomy procedure

The surgeries were performed under retrobulbar anesthesia by the same experienced ophthalmologist. Anesthesia was induced by an equal mixture of 2% lidocaine and 0.75% bupivacaine. For patients with cataract, the lens was removed via a corneal tunnel incision (3.2 mm) by continuous circular capsulorhexis, and then intracapsular phacoemulsification. A foldable intraocular lens was implanted after the vitrectomy was completed. The posterior capsular membrane was kept intact. All patients underwent a standard 3-port pars plana vitrectomy. Vitreous hemorrhage was completely cleaned and neovascular membranes were removed. The base of the vitreous body was completely removed to relieve retinal traction. Pan-retinal photocoagulation or complementary photocoagulation was performed before vitrectomy surgery for the photocoagulation group, but not in the nonphotocoagulation group.

After surgery, dexamethasone (5 mg) and antibiotics were periorbitally injected. Postoperative Tobradex eye drops were initially applied 4 times a day, with the number of applications gradually reduced when intraocular inflammation was controlled. Eight eyes had transient increase in IOP (>27 mm Hg), lasting for 1 to 5 days. Finally, the IOP was maintained at <21 mm Hg.

### 2.3 Evaluation

The IOP was measured 3 times, using an applanation tonometer. The average IOP was used for comparison. A specular microscope (KX4; Olympus, Tokyo, Japan) was used to assess the central corneal endothelium, and the measurement was made by the same examiner. The mean cell density (cell/mm^2^) and percentage of hexagonal cells (PHC) before surgery, and at 1 week, 1 month, and 3 months after surgery, were calculated.

### 2.4 Statistical analysis

Statistical analyses were performed using SPSS 19.0. Quantitative data are expressed as mean ± standard deviation. Student *t* tests were used to compare differences between the photocoagulation group and the nonphotocoagulation group. One-way analysis of variance (ANOVA) was used to compare differences in the IOP, cell density, and PHC at different timepoints within the same group. Probability values <.05 were considered statistically significant.

## 3 Results

### 3.1 Baseline characteristics

There were no significant differences in the mean age, blood pressure, fasting blood glucose concentration, serum triglyceride, or serum total cholesterol levels between the photocoagulation and nonphotocoagulation groups (Table 1). Also comparable between these 2 groups were preoperative IOP, cell density, and PHC.

**Table 1:**
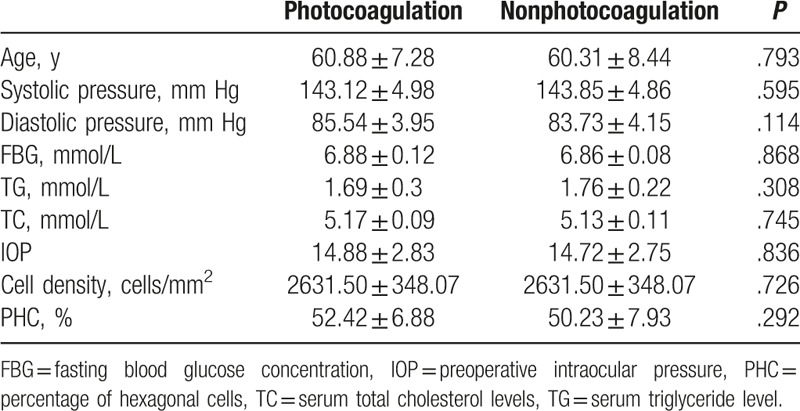
Baseline (preoperative) characteristics of patients in the photocoagulation and nonphotocoagulation groups.

### 3.2 IOP

In both the photocoagulation and nonphotocoagulation groups, the mean IOPs at 1 week, 1 month, and 3 months after surgery was not significantly different from the preoperative value (*P* > .05; Table 2). In addition, at each time point there were no significant differences in the mean IOPs between the 2 groups (*P* > .05).

**Table 2:**
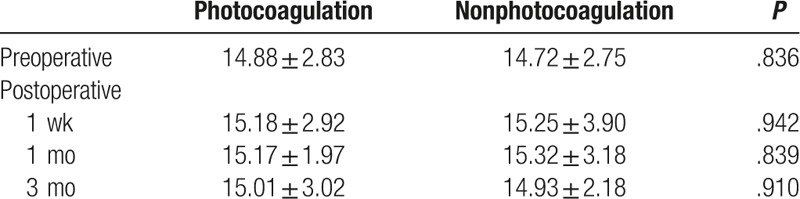
IOP in the photocoagulation and nonphotocoagulation groups (mm Hg).

### 3.3 Cell density

In the photocoagulation group, the mean cell densities at 1 week and 1 month after surgery were not significantly different from the preoperative value (*P* > .05; Table 3). The mean cell density at 3 months after surgery was significantly lower compared with the preoperative value (*P* = .01).

**Table 3:**
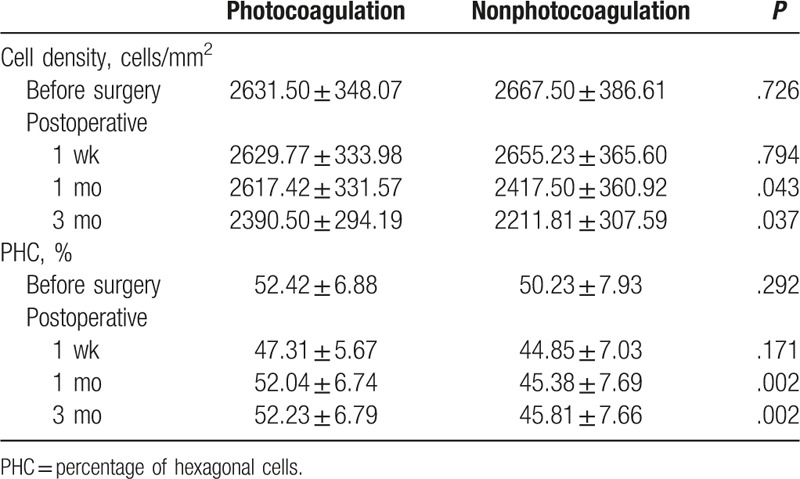
Mean cell density and PHC in the photocoagulation and nonphotocoagulation groups.

In the nonphotocoagulation group, the mean cell density at 1 week after surgery was not significantly different from the preoperative value (*P* > .05; Table 3). The mean cell densities at 1 and 3 months after surgery were significantly lower compared with the preoperative value (*P* = .02 and *P* < .001, respectively).

One week after surgery, the mean cell densities of the photocoagulation and nonphotocoagulation groups were similar (*P* > .05). However, the mean cell densities at 1 and 3 months after surgery were significantly higher in the photocoagulation group than in the nonphotocoagulation group (*P* = .043 and *P* = .037, respectively).

### 3.4 Percentage of hexagonal cells

In the photocoagulation group, the PHC at 1 week after surgery was significantly lower than the preoperative value (*P* = .005). The percentages of hexagonal cells at 1 and 3 months after surgery were not significantly different from the preoperative value (*P* > .05).

In the nonphotocoagulation group, the percentages of hexagonal cells at 1 week, 1 month, and 3 months after surgery were significantly lower than the preoperative value (*P* = .012, *P* = .03, and *P* = .046, respectively).

The PHC at 1 week after surgery in the photocoagulation group was similar to that of the nonphotocoagulation group (*P* > .05). The percentages of hexagonal cells at 1 and 3 months after surgery were significantly higher in the photocoagulation group than in the nonphotocoagulation group (*P* = .002 and *P* = .002, respectively).

## 4 Discussion

In this study, we compared the changes in corneal endothelial cells of PDR patients who received photocoagulation before vitrectomy, to those who did not receive photocoagulation. The mean cell densities and percentages of hexagonal cells at 1 and 3 months after surgery were significantly higher in patients who were treated with preoperative photocoagulation. This study suggests that photocoagulation before vitrectomy can reduce subsequent corneal endothelial cell damage in PDR patients.

To exclude the influence of age on corneal endothelial cells,^[21]^ in the present study the photocoagulation and nonphotocoagulation groups were matched for age. These PDR patient groups also had similar blood pressure, fasting blood glucose concentration, and serum triglyceride and total cholesterol levels. Thus, the systemic conditions of the patients in the 2 groups were comparable.

It has been reported that diabetic patients have structurally and functionally abnormal corneal endothelial cells that are susceptible to damage.^[11–14]^ In the present study, the preoperative mean cell densities and percentages of hexagonal cells were not significantly different between the 2 groups. This indicates that the preoperative condition of the corneal endothelial cells of the 2 groups were similar. In addition, the corneal endothelial cell density of patients with glaucoma is lower than that of healthy controls,^[22]^ suggesting that a high IOP may be a risk factor for corneal endothelial cell loss. In the present study, the IOP values at each timepoint before surgery, and 1 week, 1 month, and 3 months after surgery were similar between the 2 groups. This suggests that differences in cell density or PHC between the 2 groups are not due to IOP.

In this study, we examined the corneal endothelial cell densities and percentages of hexagonal cells before vitrectomy and at 1 week, 1 month, and 3 months after vitrectomy in PDR patients. In both the photocoagulation and nonphotocoagulation groups, at 1 week after surgery the cell densities were similar to the preoperative values, but the percentages of hexagonal cells were significantly lower. This suggests that at 1 week after surgery the corneal endothelial cell morphology had changed, although the number of corneal endothelial cells had not changed.

Reportedly, the corneal endothelial cells of diabetic patients are morphologically abnormal, and it is changes in cell morphology, but not cell number, that are the main contributor to corneal endothelial cell injury after surgery in these patients.^[13,15,23,24]^ Therefore, our study suggests that dysfunction of corneal endothelial cells occurred at 1 week after surgery in PDR patients. Watanabe et al^[25]^ found that changes in corneal thickness may be useful for evaluating the extent of surgical stress after vitrectomy. Our findings that the PHC had decreased 1 week after vitrectomy suggests that surgical stress induced by vitrectomy may cause corneal endothelial cell damage.

Anterior chamber inflammation commonly occurs after vitrectomy^[26]^ and can cause corneal endothelial cell damage. We found that in the photocoagulation group, at 3 months after surgery the cell density had begun to decrease, while in the nonphotocoagulation group, the cell density was reduced at 1 month. In the patients who underwent photocoagulation, the cell densities at 1 and 3 months were significantly higher than that of the nonphotocoagulation group. These findings suggest that photocoagulation before vitrectomy can reduce subsequent corneal endothelial cell loss in PDR patients. In addition, at 1 month after surgery the PHC in the photocoagulation group were similar to preoperative values, but this was not true of the nonphotocoagulation group. This suggests that photocoagulation before vitrectomy can promote subsequent recovery of corneal endothelial cell morphology in PDR patients.

## 5 Conclusion

In this study, we found that PDR patients with previtrectomy photocoagulation were associated with subsequent less reduction in corneal endothelial cell density and PHC, compared with those in patients who did not undergo photocoagulation before vitrectomy surgery. Preoperative photocoagulation may reduce inflammation and surgical stress caused by vitrectomy, thus reducing corneal endothelial damage. In addition, preoperative photocoagulation may promote recovery of corneal endothelial cell morphology. Taken together, our findings suggest that photocoagulation performed before vitrectomy may reduce subsequent corneal endothelial cell damage and PHC decrement in PDR patients with vitreous hemorrhage.
